# Foodborne Pathogens Recovered from Ready-to-Eat Foods from Roadside Cafeterias and Retail Outlets in Alice, Eastern Cape Province, South Africa: Public Health Implications

**DOI:** 10.3390/ijerph9082608

**Published:** 2012-07-27

**Authors:** Mirriam E. Nyenje, Collins E. Odjadjare, Nicoline F. Tanih, Ezekiel Green, Roland N. Ndip

**Affiliations:** 1 Department of Biochemistry and Microbiology, Faculty of Science and Agriculture, University of Fort Hare, PMB X1314, Alice 5700, South Africa; Email: nyenjem@yahoo.com (M.E.N.); ecodjadjare@yahoo.com (C.E.O.); nicofriline@yahoo.com (N.F.T.); egreen@ufh.ac.za (E.G.); 2 Department of Microbiology and Parasitology, Faculty of Science, University of Buea, P.O. Box 63, Buea, Cameroon

**Keywords:** street foods, microbial quality, foodborne pathogens, bacterial count, South Africa

## Abstract

This study assessed the microbiological quality of various ready-to-eat foods sold in Alice, South Africa. Microbiological analysis was conducted on 252 samples which included vegetables, potatoes, rice, pies, beef and chicken stew. The isolates were identified using biochemical tests and the API 20E, API 20NE and API Listeria kits; results were analyzed using the one-way-ANOVA test. Bacterial growth was present in all the food types tested; high levels of total aerobic count were observed in vegetables, 6.8 ± 0.07 followed by rice, 6.7 ± 1.7 while pies had the lowest count (2.58 ± 0.24). Organisms isolated included: *Listeria* spp. (22%), *Enterobacter* spp. (18%), *Aeromonas hydrophila* (12%), *Klebsiella oxytoca* (8%), *Proteus mirabilis* (6.3%), *Staphylococcus aureus* (3.2%) and *Pseudomonas luteola* (2.4%). Interestingly, *Salmonella* spp. and *Escherichia coli* were not isolated in any of the samples. There was a statistically significant difference (*p* < 0.05) in the prevalence of foodborne pathogens from hygienic and unhygienic cafeterias. The results indicated that most of the ready-to-eat food samples examined in this study did not meet bacteriological quality standards, therefore posing potential risks to consumers. This should draw the attention of the relevant authorities to ensure that hygienic standards are improved to curtain foodborne infections.

## 1. Introduction

Foodborne diseases are an increasingly recognized problem involving a wide spectrum of illnesses caused by bacterial, viral, parasitic or chemical contamination of food. Although viruses account for half of all the foodborne illnesses, most hospitalizations and deaths related to foodborne infections are due to bacterial agents. Diarrheal diseases are the commonest manifestation of food poisoning and in some cases, can lead to death. The diseases are caused by either toxin from the “disease-causing” microbe, or by the human body’s reactions to the microbe itself [1].

Street sold foods are appreciated for their unique flavors and convenience. They also assure food security for low income urban population and livelihood for a significant proportion of the population in many developing countries [2]. However, the unhygienic conditions in which these foods are prepared, stored and served raise a question regarding their microbiological quality. Researchers have investigated the microbiological quality of street vended foods in different countries; high bacterial counts and a high incidence of foodborne pathogens in such foods have been reported. In Ghana, bacterial counts of 5.13–6.36 log_10_ CFU g^−^^1^ were documented in the street foods of Kumasi [3]. Another study in Accra reported a total bacterial count range of 0.8–6.3 log_10_ CFU g^−1^, *Enterobacteriaceae* count of 0.3–4.7 log_10_ CFU g^−1^ and 0.3–3.7 log_10_ CFU g^−1^ of *Staphylococcus aureus* [4]. In Egypt, Ismail [5] studied the microbial quality of ready-to-eat meat sandwich and reported aerobic plate counts, *Enterobacteriaceae*, and *Enterococci* counts range of 2 × 10^3^–4 × 10^6^, 6 × 10–8 × 10^2^ and 3 × 10^3^–6 × 10^5^ CFU g^−1^ respectively.

Contamination of food by enteric pathogens can occur from the farm if human sewage is used to fertilize the soils or if sewage water is used to irrigate the crops. Such risks are further increased if the food is mishandled during processing and preparations where pathogens could multiply exponentially under favorable conditions [2]. However, no study has been conducted on the microbial quality of the food supplied by local food establishments in Alice. Therefore, the present study was carried out to assess the microbiological quality of various ready-to-eat foods supplied in Alice, in a bid to throw more light on the inherent risk associated with such foods. 

## 2. Results and Discussion

### 2.1. Bacterial Count of Isolates in the Food Samples

The mean bacterial count of the isolates in the food samples were expressed as log_10_ CFU g^−1^ for easy computation. Food were classified as acceptable if the bacterial count was less than or equal to 5 log_10_ CFU g^−1^ [6]. The mean value of aerobic bacterial count on vegetables, rice, potatoes, beef, chicken stew and pies were 6.8 ± 0.07, 6.7 ± 1.7, 6.27 ± 0.18, 5.32 ± 3.14, 6.05 ± 0.12 and 2.58 ± 0.24 log_10_ CFU g^−1^ respectively (Table 1). The bacterial count of vegetables, rice, potatoes, beef and chicken stew was statistically significant when compared with pies (*p* < 0.05). A similar comparison was made for food from hygienic and unhygienic sources; the results revealed that there was statistically significant difference in the bacterial load of beef stew and rice from hygienic and unhygienic cafeterias (*p* < 0.05). However, no significance was observed for vegetables, chicken and potatoes samples (*p* > 0.05) (Table 2). 

Plate count of aerobic mesophilic microorganisms found in food is one of the microbiological indicators for food quality. The presence of aerobic organisms reflects existence of favorable conditions for the multiplication of microorganisms. In this study, all the sample types tested had mean contamination levels of ≥5.0 log_10_ CFU g^−1^ except the pies. The New South Wales (NSW) Food Authority [6] recommends the standard limit for bacterial count of fully cooked ready-to-eat foods to be <5.0 log_10_ CFU g^−1^. Hence these foods could be of high risk in transmitting enteric pathogens. These findings corroborate previous works [4,7,8]. In their study, Mensah *et al*. [4] found a bacterial count of 6.3 ± 0.78 in salads sold on the streets of Accra. Likewise, Christison *et al*. [8] also reported high bacterial prevalence in filled baguettes and salads. 

Vegetables had the highest bacterial count, of 6.3–6.8 log_10_ CFU g^−1^. The findings are in agreement with other studies in Egypt, Turkey and Taiwan. Saddik *et al*. [9] reported the upper limit of 6.69 log_10_ CFU g^−1^ aerobic counts of microorganisms on minimally processed vegetable samples in Egypt while Fang *et al*. [10] reported an aerobic plate count on salad vegetable samples in Taiwan ranging from 3.30–8.64 log_10_ CFU g^−1^. Furthermore, Vural and Erkan [11] in Turkey had a range of aerobic plate counts from 6.43 to 7.63 log_10_ CFU g^−1^. 

Vegetables have been associated with foodborne outbreaks in many countries; they may be contaminated from the farm with human sewage, and from the irrigation water. Unsafe water used for rinsing the vegetables and sprinkling to keep them fresh are other possible sources of contamination [7]. As most of these produce are eaten raw or with minimal cooking, their microbial content may represent a risk factor for the consumer’s health [12]. Some of these factors might be the possible source of contamination in the vegetables under study. A study in Morocco, reported the occurrence of pathogenic bacteria mostly of the *Enterobacteriaceae* family in vegetables irrigated by untreated wastewater. Although usually regarded as human pathogens, members of this family have also been recognized as inhabitants of soil and plants. Thus, vegetables may serve as a reservoir from which these bacteria can colonize and infect a susceptible host [13].

Worthy of note is the fact that unhygienic cafeterias registered high bacterial counts; lack of sources of running water, refrigeration facilities, and post production operations and personal hygiene of the food handlers might be the possible contributing factors. It was also observed that in the unhygienic shops, vendors washed the utensils and dishes used for preparation and serving their food in buckets containing unclean water which were likely not replaced throughout the whole day. This might also be another reason for cross-contamination.

**Table 1:** Mean bacterial counts of the food samples examined.

Food types	Bacterial count range (log _10_ CFU g^−1^)	Mean bacterial count (log _10_ CFU g^−1^) ± SD	*p*-value					
			V	R	C	B	PT	P
Vegetables (n = 42)	6.3–6.8	6.8 ± 0.07	-	0.235	0.913	0.001	0.585	0.000
Rice (n = 42)	4.3–6.7	6.7 ± 1.7	0.235	-	0.196	0.000	0.513	0.000
Chicken stew (n = 42)	5.9–6.2	6.05 ± 0.12	0.913	0.196	-	0.001	0.513	0.000
Beef stew (n = 42)	3.9–6.15	5.32 ± 3.14	0.001	0.000	0.001	-	0.000	0.000
Potatoes (n = 42)	6.0–6.4	6.27 ± 0.18	0.585	0.513	0.513	0.000	-	0.000
Pies (n = 42)	2.30–2.81	2.58 ± 0. 24	0.000	0.000	0.000	0.000	0.000	-

CFU g^−1^, colony forming units per gram; SD, standard deviation; R, rice; C, chicken stew; B, beef stew; PT, potatoes; P, pies; -, no comparison done. The mean difference is considered significant at *p* < 0.05.

**Table 2:** Mean bacterial counts of food obtained from hygienic and unhygienic cafeterias.

Food types	Bacterial count (log _10_ cfu/g) ± Standard deviation			*p*-value
	Unhygienic		Hygienic	
Vegetables (n = 42)	6.8 ± 0.07	6.4 ±0.7		0.128 (>0.05)
Rice (n = 42)	6.7 ± 1.7	6.35 ± 0.07		0.000 (<0.05)
Chicken stew (n = 42)	6.05 ± 0.12	5.95 ± 0.07		0.122 (>0.05)
Beef stew (n = 42)	5.32 ± 3.14	4.32 ±3.01		0.002 (<0.05)
Potatoes (n = 42)	6.27 ± 0.18	6.15 ± 0.21		0.171 (>0.05)
Pies (n = 42)	ND	3.9 ± 0.7		ND

CFU g^−1^, colony forming units per gram; ND, not determined; unhygienic cafeterias, vending sites without running water, toilets, fridges to store food and dirt environment; hygienic cafeterias, vending sites with running water, clean food preparation surfaces, toilets, clean environment and food handlers who comply with food hygienic standards. The mean difference is considered significant at *p* < 0.05.

### 2.2. Prevalence of Foodborne Pathogens in the Various Food Types

**Figure 1:**
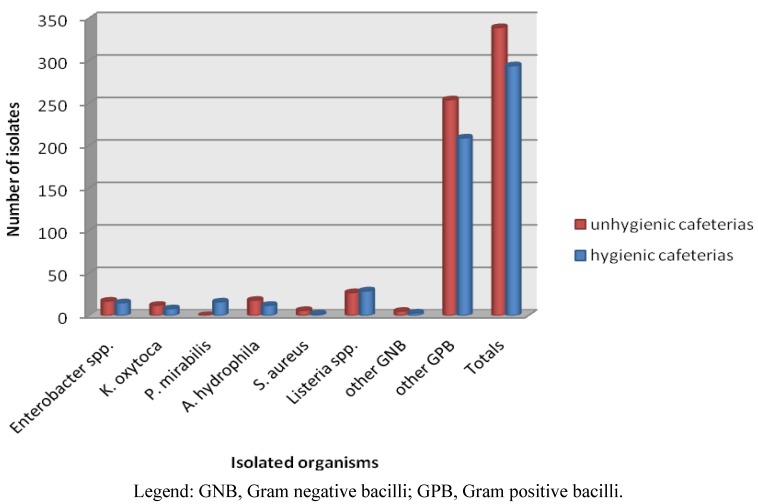
Bacterial contamination of food from hygienic and unhygienic cafeterias.

Table 3 depicts the occurrence of possible pathogens in the 252 food samples tested. Bacterial growth was observed in all the food types; the most prevalent bacteria were *Listeria* spp. (22%), *Enterobacter* spp. (18%), *Aeromonas hydrophilla* (12%), *Klebsiella oxytoca* (8%) and *Proteus mirabilis* (6.3%). Vegetables and rice had the highest level of contamination with 108 (18%) isolates each. *Listeria ivanovii* was prevalent in pies (33%) followed by chicken (28%) while beef, rice and potatoes registered 14% each. *Enterobacter cloacae* was mainly isolated from beef stew (24%), pies and chicken (17%) each and rice 14%. *Aeromonas hydrophila* was detected in 10 (24%) of the vegetables, seven (17%) of rice and six (14%) of potatoes. *Klebsiella oxytoca* was present in seven (17%) of pies and potatoes, and in six (14%) of vegetables. It was also observed that the prevalence of the pathogens in the environment varied as *Proteus mirabilis* was isolated from seven (17%) of rice, four (10%) of chicken and three (7%) of beef obtained from one of the university cafeterias while none was isolated from the other shops. Interestingly, *E. coli* and *Salmonella* spp. which are common food pathogens were not isolated. The prevalence of foodborne pathogens from hygienic and unhygienic cafeterias was also compared. The unhygienic cafeterias recorded the highest number of isolates 339 (54%) compared to 299 (47%) from hygienic cafeterias (Figure 1). However, *L. ivanovii* and *P. mirabilis* were the only isolates which were recovered more from unhygienic cafeterias than hygienic cafeteria. 

**Table 3:** Bacteria distribution in the various food samples examined.

Bacteria isolates	Chicken stew (n = 42)	Beef stew (n = 42)	Vegetables (n = 42)	Rice (n = 42)	Potatoes (n = 42)	Pies (n = 42)	Number (%) occurrence
*Listeria ivanovii*	11	6	8	6	6	14	51/252 (20%)
*Listeria grayi*	1	0	1	1	1	1	5/252 (2%)
*Enterobacter cloacae*	7	10	2	6	3	7	35/252 (14%)
*Enterobacter sakazaki*	1	0	4	0	1	0	6/252 (2.4)
*Enterobacter gergoviae*	0	0	0	0	0	2	2/252 (1%)
**Enterobacter cancerogenus**	0	0	1	0	1	1	3/252 (1.2%)
*Proteus mirabilis*	4	3	2	7	0	0	16/252 (6.3%)
*Klebsiella oxytoca*	2	2	6	1	7	3	21/252 (8.3%)
*Aeromonas hydrophila*	2	4	10	7	6	0	30/252 (12%)
*Staphylococcus aureus*	1	0	0	2	3	2	8/252 (3.2%)
*Pantoea* spp.	0	3	2	0	2	0	7/252 (2.8%)
**Pseudomonas luteola**	0	1	2	1	0	2	6/252 (2.4%)
Other Gram positive Bacilli	54	62	70	77	64	66	267/588 (45%)
**Total isolates**	**85**	**93**	**108**	**108**	**94**	**100**	**588**

The study found that ready-to-eat foods from roadside cafeterias contained more organisms if compared to that sold in standard fast foods centres and supermarkets which had running water and cement floors. Bukar *et al*. [7] found that ready-to-eat rice sold on the streets of Kano contained more microorganisms if compared to that sold in standard fast food centres. The road side cafeterias had no running water, hands and utensils washing was done in one bucket where the water was not regularly changed. This coupled with unhygienic surroundings like sewage, improper waste disposal system, might be the possible sources of food contamination in these sites.

Members of the family *Enterobacteriaceae* have been considered a potent cause of foodborne outbreaks [14]. In this study, *Enterobacteriaceae* represented 46% of the isolates. *Enterobacter cloacae* (14%) was the most prevalent among the isolated *Enterobacteriaceae*. The findings concurs the work of Falomir *et al*. [12] who found *Enterobacter cloacae* and *Klebsiella oxytoca*, to be the most prevalent coliforms in ready-to-eat-salads served in the dining halls of a pre-school and a primary school in Valencia city, Spain. *Enterobacter* spp. are the sixth most common cause of nosocomial infection in particular, *Enterobacter cloacae* have been implicated in a broad range of clinical syndromes. The literature is replete with descriptions of bacteraemia, meningitis, urinary tract infection, septicaemia, wound infection, central nervous system and gastrointestinal tract infections [2]. Therefore the presence of these organisms in the foods under study might pose a health risk to children and individuals with underlying conditions. 

*Listeria* species has been associated with a wide variety of food sources particularly poultry, red meat and meat products [15]. The present study recorded a high occurrence of *Listeria* spp. in pies (33%) and chicken stew (28%). These findings are in line with those of other authors [15,16,17]. In Egypt, *Listeria* contamination rate in meat and chicken products was reported to be 41% [15], lower than the 73.9% reported in Malaysia from imported frozen beef [18] and 83.3% from raw minced meat in Turkey [17]. The bacteria can be endemic in food processing environments, because it survives food-processing technologies that rely on acidic or salty conditions and, unlike many pathogens, can continue to multiply slowly at low temperatures, allowing for growth even in properly refrigerated foods. Hence, their presence may be indicative of poor hygiene or cross contamination, which is considered to be a possible source of *Listeria* contamination in processed meat [19]. In addition, minced/chopped meat products by their nature, undergo extensive processing and handling during their production thereby increasing the risk of contamination. This might explain the high prevalence of *L.ivanovii* in pies which were of either minced/chopped chicken or red meat.

Nevertheless, despite the high rates of contamination of certain foods with *Listeria* species, listeriosis is a relatively rare disease as compared with other common foodborne illnesses. However, because of its high case fatality rate of approximately 20–30%, listeriosis has been ranked second, after salmonellosis as the most frequent cause of foodborne infection-related deaths in Europe [19]. 

Clinical manifestations of listeriosis range from febrile gastroenteritis to more severe invasive forms, including sepsis, meningitis, rhombencephalitis and perinatal infections. Perinatal listeriosis can lead to abortion, birth of a stillborn fetus or a baby with generalized infection (granulomatosis infant-septica), and sepsis or meningitis in the neonate [20].

*Listeria monocytogenes* is the most common causative agent of human listeriosis. However, *L.* *ivanovii*, an animal pathogen has been previously isolated, although rarely, from infected humans, indicating pathogenic potential for humans [20]. Therefore the isolation of *L. ivanovii* in the present study might reflect a health risk to the consumers particularly pregnant women. 

The study registered a low *Proteus mirabilis* occurrence of 6.3%. It is noteworthy that all 16 isolates were obtained from one source (one of the university shops). *Proteus mirabilis* is a proteolytic bacterium implicated in food deterioration and spoilage. In addition to opportunistic infections, it can also cause food poisoning when consumed in contaminated food such as meat, vegetables, and seafood [21].

*Staphylococcus aureus* was prevalent in 3.2% of the samples. The results were contrary to the findings of Ghosh *et al*. [2] and Kumar *et al*. [22] who reported high prevalence of *Staphylococcus aureus* in street-vended foods. Differences in geographical location and personal hygiene of the food handlers might help to explain this discrepancy. In their study, Ghosh *et al.* [2] reported high prevalence of *Staphylococcus aureus* from coriander sauce 91 (60%) and 129 (86%) from ready-to-eat salads in India. The authors attributed this high prevalence to poor hygienic conditions of the premises due to rubbish, sewage and other noxious substances present in the vicinity. Kumar *et al.* [22] presented the high staphylococcal count from fruit chaat (a drink made from a mixture of fruits and vegetables) samples obtained from mobile vendors without any covering compared to those samples from mobile vendors with coverings. The authors suggested that, covering to some extent acts as a shield for air borne bacterial pathogens. 

*Staphylococcus* species are found on the skin and in the nose and throat of most healthy people; they are also widespread in untreated water, raw milk and sewage. When *Staphylococcus aureus* is allowed to grow in foods, it can produce a toxin that causes illness. Although, cooking destroys the bacteria, the toxin produced by *Staphylococcus aureus* is heat stable and may not be destroyed even by heating, let alone by refrigeration. Foods that are handled frequently during preparation are prime targets for *Staphylococci* contamination [23].

The prevalence of *A. hydrophila* was at 12% with the organism isolated more from vegetables (10/42) than in the other food types. Other studies reported a prevalence of 4%, 26% and 41% Aeromonads in dairy products, vegetables and sea foods respectively [24,25]. Although *A. hydrophila* is water based, waterborne outbreaks have not been reported, and waterborne transmission has not been well established as various studies have been unsuccessful in linking patient isolates of *A. hydrophila* with isolates recovered from the water supply [26,27]. Nevertheless, this bacterium has gained public health recognition as an opportunistic pathogen. It has been implicated as a potential agent of gastroenteritis, septicaemia, cellulitis, colitis, and meningitis, and is frequently isolated from wound infections sustained in aquatic environments [28,29]. Palumbo *et al*. [30] found *Aeromonas* isolates universally present in all foods tested, including sea-foods, raw milk, chicken, and meats such as lamb, veal, pork, and ground beef. 

## 3. Experimental Section

### 3.1. Study Area

Alice is a rural settlement in the Nkonkobe municipality of the Eastern Cape province of South Africa and situated in the geographical coordinates 32°50’36’’ S, 26°55’00” E; with a population of about 9,788 and a student population of 8,548 [31]. The study was conducted between August and November, 2011. Two university restaurants and eight ready-to-eat food vending sites in Alice Town were sampled. These sites were chosen because they are very popular among students, workers, commuters, shoppers and passers-by. Members of these groups buy food from at least one of these out lets at one time or the other. They include two garages that were designated (G1 and G2), three supermarkets (S1, S2 and S3), four roadside cafeterias and two university restaurants (C1, C2, C3, C4, C5 and C6). The sites were classified as unhygienic cafeterias if they had no running water, poor hygienic conditions of the staff and surrounding *i.e.*, rubbish, sewage and other noxious substances present in the vicinity that can attract foodborne vectors such as flies, whereas hygienic cafeterias were termed those with running water, clean food preparation surfaces, toilets, clean environment and food handlers who comply with food hygienic standards. CAC/GL 22R [32], defined food hygiene as all conditions and measures necessary to ensure the safety and suitability of food at all stages.

### 3.2. Sample Collection

A total of 252 cooked samples were purchased comprising of 42 batches of six food types which included beef and chicken stew, potatoes, rice, vegetables and pies all of which are popular foods from cafeterias in the study area. Samples were packed separately and transported to the Microbial Pathogenicity and Molecular Epidemiology Research Laboratory of the University of Fort Hare for immediate processing. 

### 3.3. Bacteriological Analysis of the Samples

#### 3.3.1. Sample Preparation, Culture and Bacterial Count

Samples were prepared according to the method of Akoachere *et al*. [33] with some modifications. Twenty five grams of each sample was weighed and homogenized by blending in 225 mL of sterile buffered peptone water. One millilitre of the homogenate was introduced into 9 mL of the buffered peptone water in a test tube, labelled 1:10 (10^−1^) dilution and serially diluted to five other test tubes labelled 10^−2^, 10^−3^, 10^−4^, 10^−5^ and 10^−6^; the procedure was repeated for each sample and the blender was carefully cleaned and disinfected in between samples to prevent any cross contamination. One hundred microliters of each of the diluted sample was plated on Nutrient agar. The plates were incubated aerobically for 24 h at 37 °C. All discrete colonies were counted where possible and expressed as the log_10_ of colony forming units per gram (CFU g^−^^1^). To improve recovery and detection, the tubes were incubated aerobically at 37 °C for 12–24 h after which a loopful of enrichment broth was cultured on Salmonella/Shigella, Mac Conkey, Eosin-Methylene Blue (EMB) and Colombia blood agar (Oxoid, Basingstoke, England) supplemented with 5% horse blood. The plates were incubated aerobically for 24–48 h at 37 °C. 

#### 3.3.2. Isolation and Biochemical Characterization of the Isolates

Colonies were presumptively identified by colony pigmentation and Gram staining characteristics. Pure cultures were obtained by streaking a portion of an isolated colony on nutrient agar and incubated aerobically at 37 °C for 24 h. The isolates were confirmed by oxidase, catalase, and coagulase activity. Isolates were further characterized biochemically using API 20E whilst API 20NE was used for the identification of non-fastidious and non-enteric Gram negative rods. The confirmation of *Listeria* spp. was carried out using API Listeria kit (Biomerieux, Marcy-L’etoile, France). The tests were performed according to manufacturer’s instruction for use. Briefly, a single colony from young cultures (18–24 h) was emulsified in 5 mL of sterile sodium chloride (0.85%) and the turbidity adjusted to the equivalent of the turbidity of 0.5 McFarland standards. The standardized bacterial suspension was distributed into the tubes of the test strip carefully to avoid the formation of bubbles. Anaerobiosis was created by overlaying with sterile mineral oil; the strips were then incubated in humid atmosphere for 18–24 h at 37 °C. Data interpretation was performed using the Analytical profile index (API) database (V4.1) with the apiweb^TM^ identification software.

## 4. Statistical Analysis

Statistical analysis was performed using excel and SPSS version 19. The bacterial counts were expressed as mean ± Standard deviation using excel. One way ANOVA followed by Turkey’s *post hoc* test was used to compare the bacterial counts in various food types and bacterial count from hygienic and unhygienic cafeterias. The mean difference was considered significant at *p* < 0.05.

## 5. Conclusions

These findings demonstrate that ready-to-eat food sold in Alice Town constitutes a likely potential hazard to human health. The isolation of *Enterobacteriaceae* in ready-to-eat foods that are fully cooked is a good indicator of post-processing contamination or inadequate cooking. Therefore access to running water and health education to the vendors on personal hygiene, food safety and proper disposal of waste would improve food quality thereby reducing food borne incidences.
